# Supplementary material for: Sensitive quantification of carbon monoxide in vivo reveals a protective role of circulating hemoglobin in CO intoxication
Source: Commun Biol. 2021 Mar 29;4:425. doi: 10.1038/s42003-021-01880-1 (PMC8007703; doi:10.1038/s42003-021-01880-1)
Supplement: Supplementary file 2 — Supplementary Information [file 42003_2021_1880_MOESM2_ESM.pdf]

Supplementary Information for

Sensitive quantification of carbon monoxide *in vivo* reveals a protective role of circulating hemoglobin in CO intoxication

Qiyue Mao, Akira T. Kawaguchi, Shun Mizobata, Roberto Motterlini,* Roberta Foresti,* and Hiroaki Kitagishi*

*Corresponding authors. Email: roberto.motterlini@inserm.fr (RM), roberta.foresti@inserm.fr (RF), hkitagis@mail.doshisha.ac.jp (HK)

This PDF file includes:

Fig. S1. Stabilities of CO-hemoCD1 and CO-Hb upon N_2 bubbling.
Fig. S2. Stabilities of CO-hemoCD1 and CO-Hb against oxidation.
Fig. S3. Stabilities of CO-, deoxy-, and oxy-hemoCD1 in the solution containing biocomponents from cell lysates.
Fig. S4. Ligand exchange of CO-Hb and oxy-hemoCD1.
Fig. S5. Stabilities of CO-hemoCD1 and CO-Hb against NO.
Fig. S6. Effect of Py3CD and hemoCD1 on cell viability.
Fig. S7. Quantification of endogenous CO in tissues.
Fig. S8. Plots of the wet weight of tissues versus the amount of CO detected by the hemoCD1 assay.
Fig. S9. Effects of external reactive species, pH, and light on quantification of endogenous CO in the liver and brain tissues by hemoCD1.
Fig. S10. Comparison between the hemoCD1 assay and the gas chromatography method for CO quantification in tissues.
Fig. S11. Schematic representation of the experimental setup for CO gas inhalation in rats.
Fig. S12. *In vitro* experiments to demonstrate CO transfer from tissues to Hb.
Fig. S13. *In vitro* experiments to monitor CO transfer from cells to Hb in medium.
Fig. S14. UV-vis spectra of rat urine collected after i.v. infusion of oxy-hemoCD1 to CO-treated rats.
Fig. S15. Effect of air/O_2 ventilation in combination with hemoCD1 on CO distribution in tissues after CO inhalation *in vivo*.
Fig. S16. Effect of O_2 ventilation in combination with hemoCD1 on CO distribution in tissues after CO inhalation *in vivo*.

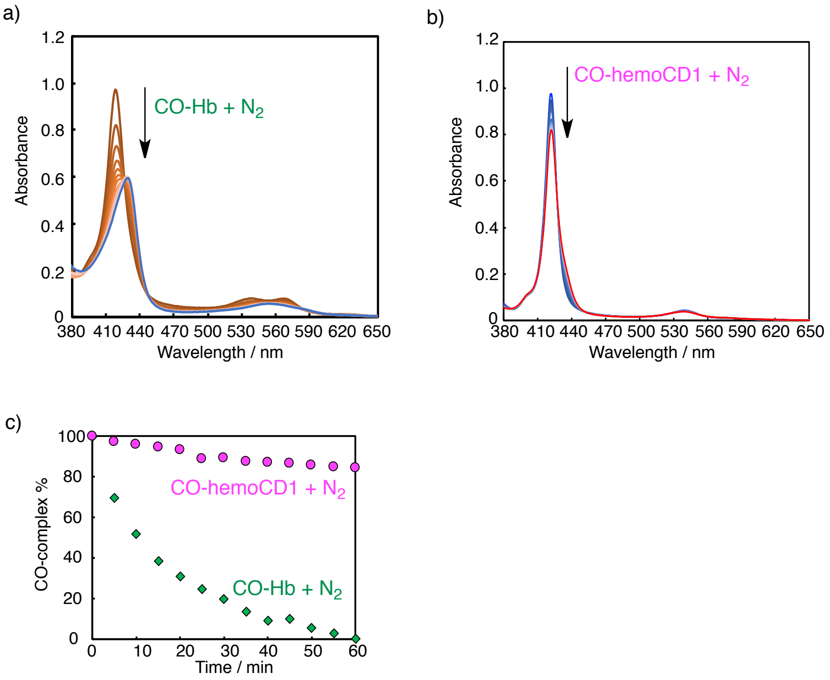

Fig. S1. **Stabilities of CO-hemoCD1 and CO-Hb upon N_2 bubbling.** (a,b) UV-vis spectral changes of CO-Hb (a) and CO-hemoCD1 (b) in phosphate buffer saline (PBS) at pH 7.4 and 25°C after N_2 bubbling. (c) The plot of the residual CO-complexes (%) of Hb and hemoCD1 during N_2 bubbling. The data indicate that CO-Hb was converted to its deoxy-form by reducing CO partial pressure, while CO-hemoCD1 was hardly affected due to its high CO binding affinity and stability.

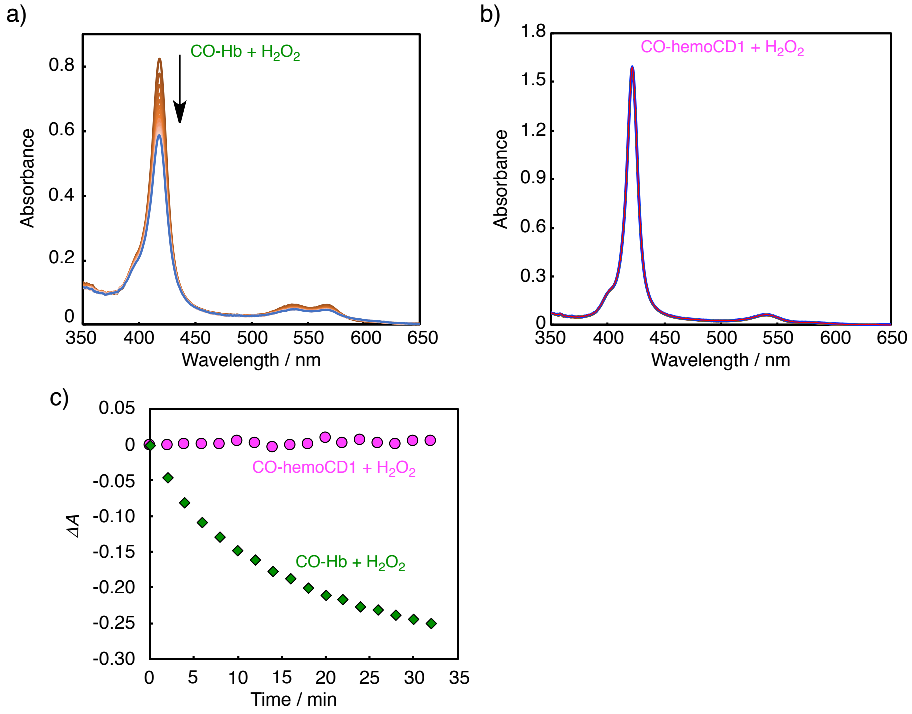

Fig. S2. **Stabilities of CO-hemoCD1 and CO-Hb against oxidation.** (a,b) UV-vis spectra of CO-Hb (a) and CO-hemoCD1 (b) in phosphate buffer saline (PBS) at pH 7.4 and 25°C after the addition of 20 eq of H_2O_2 at 2 min recording time intervals. (c) The plot of the changes in absorbances at λ_{max} of CO-Hb (418 nm) and CO-hemoCD1 (422 nm). The data indicate that CO-Hb was gradually oxidized and decomposed by H_2O_2, while CO-hemoCD1 was resistant to oxidation by H_2O_2.

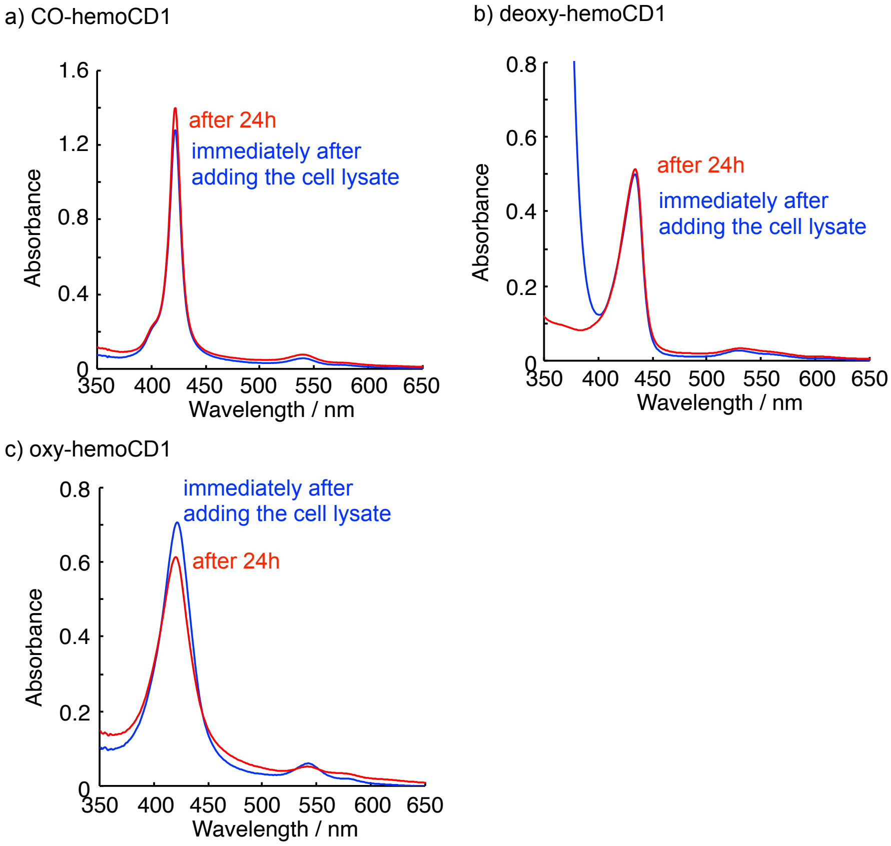

Fig. S3. Stabilities of CO-, deoxy-hemoCD1 and oxy-hemoCD1 in the solution containing biocomponents from cell lysates (10^6 cells). The spectra were measured at 25°C immediately after adding the cell lysate and after 24 h. Deoxy- and CO-hemoCD1 were quite stable over 24 h while oxy-hemoCD1 was gradually autoxidized to ferric met-form in the same manner as in the absence of cell lysates.

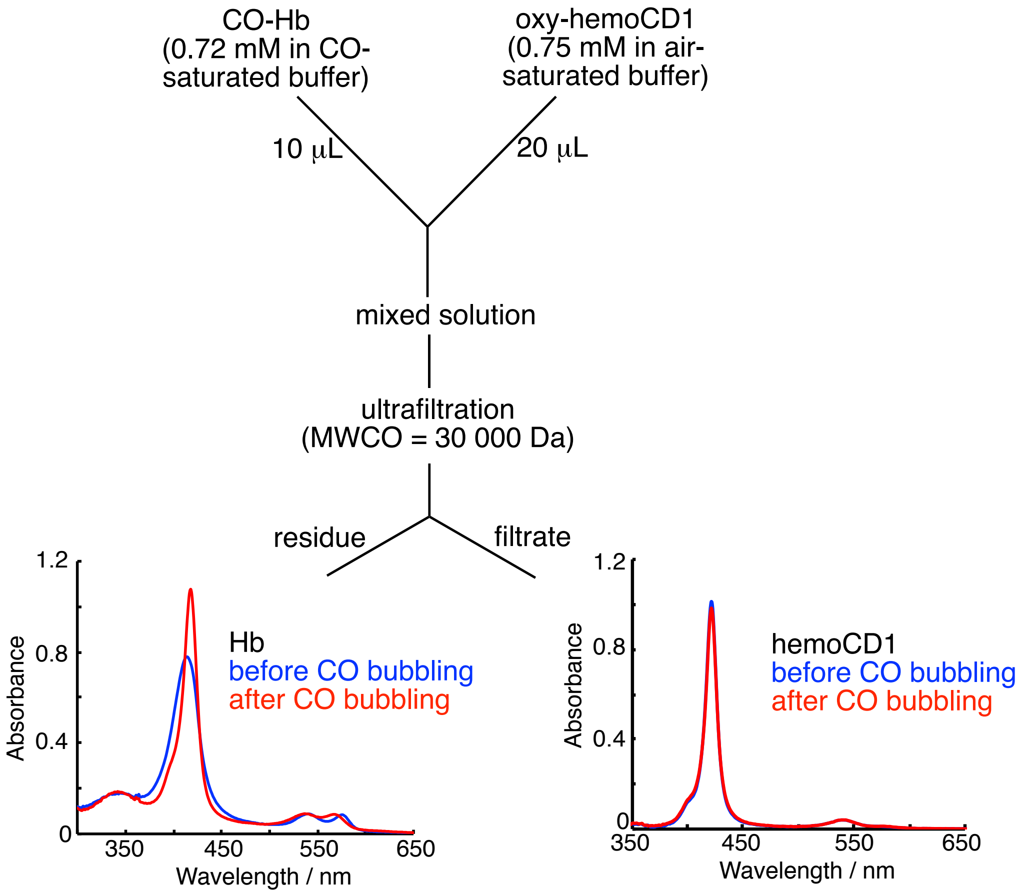

Fig. S4. An experiment for ligand exchange of CO-Hb and oxy-hemoCD1 using an ultrafilter unit. Two solutions contained CO-Hb (0.72 mM in CO-saturated buffer) and oxy-hemoCD1 (0.75 mM in air-saturated buffer) were mixed then filtered using an ultrafilter with molecular weight cut off = 30 000 Da (Amicon Ultra). The resulting residue and filtrate were appropriately diluted with PBS for UV-vis measurement. CO gas was bubbled to each solution after the measurement. The spectral characteristics indicate the residue containing significant amount of oxy-Hb and the filtrate mostly containing CO-hemoCD1, as a result of quantitative ligand exchange between each other.

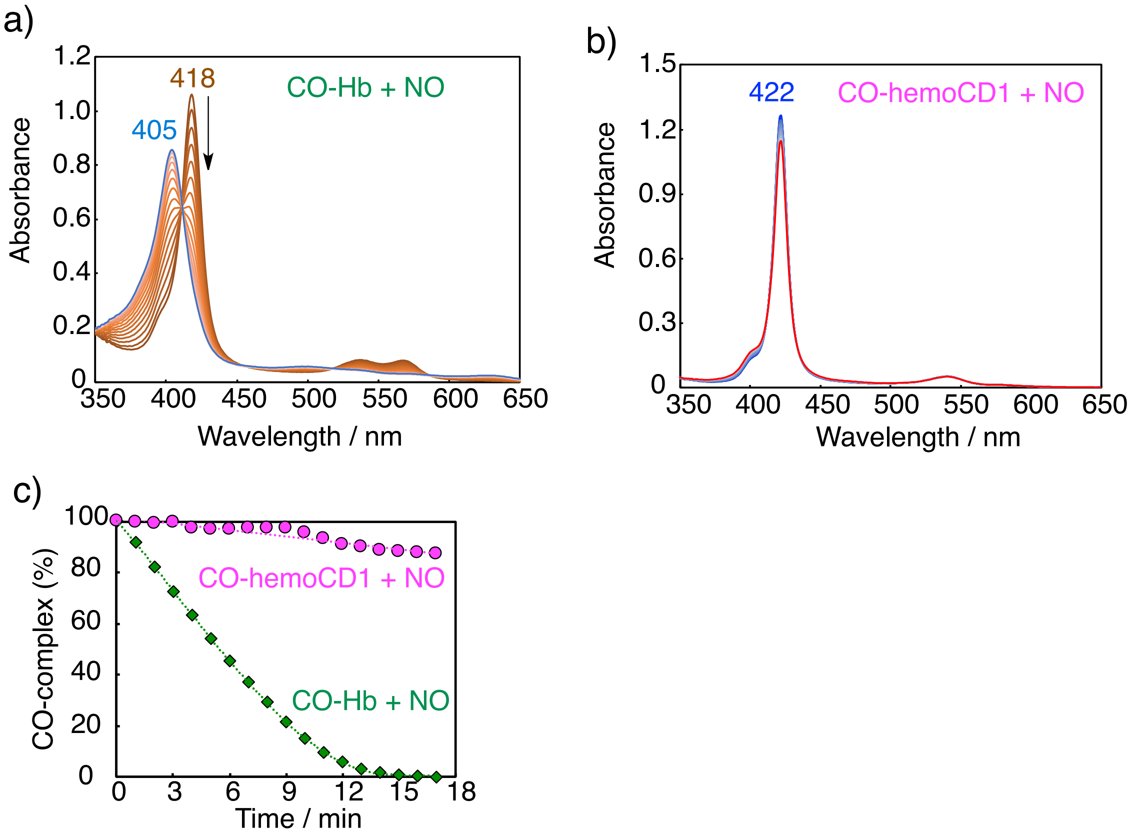

Fig. S5. **Stabilities of the CO-complexes of Hb and hemoCD1 against NO.** (a,b) UV-vis spectra of CO-Hb (a) and CO-hemoCD1 (b) after addition of NO (2.5 eq). NO was added as NOC (1-hydroxy-2-oxo-3-(3-aminopropyl)-3-isopropyl-1-triazene), a NO donor purchased from Dojin Chemicals. (c) The plot of the residual CO-complexes (%) of Hb and hemoCD1 during the reaction with NO. The data indicate that CO-Hb was smoothly converted to its met-form by the addition of NO, as we reported in a previous paper (H. Kitagishi et al., *J. Am. Chem. Soc.* **2016**, *138*, 5417, Supporting Information). In the case of CO-hemoCD1, its spectra were almost unchanged after reaction with NO due to the high stability of the CO-complex. This indicates that the CO binding affinity to hemoCD1 is much higher than that for NO. In addition, as we discussed in *J. Am. Chem. Soc.* **2017**, *139*, 5984 (Supporting Information), hemoCD1 cannot scavenge NO from NO-Hb due to its lower NO affinity compared to Hb. This indicates that NO does not affect the CO-scavenging ability of hemoCD1.

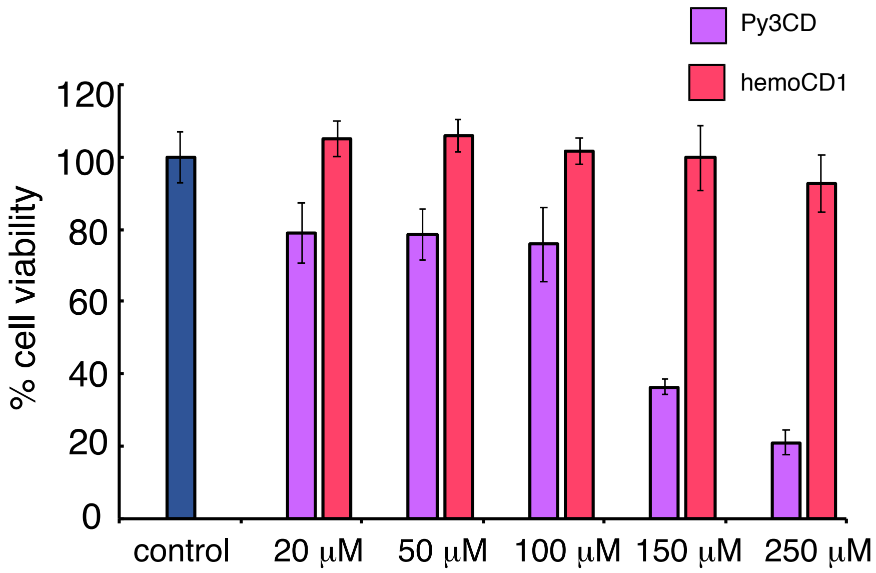

Fig. S6. **Effect of Py3CD and hemoCD1 on cell viability.** Hepatocytes were incubated with different concentrations of Py3CD or met-hemoCD1 for 3 h. Cell viability was measured using an MTT assay. Each bar represents the mean ± SE (*n* = 6).

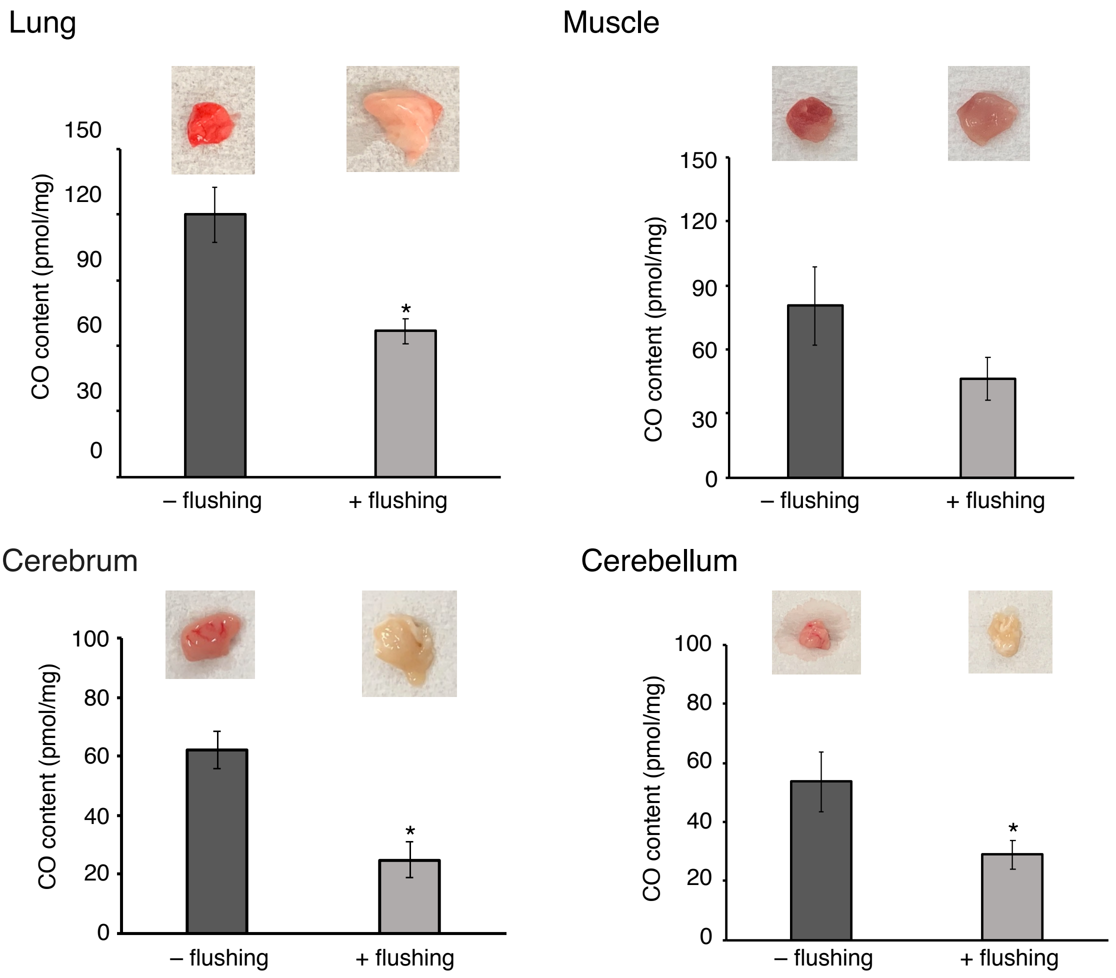

Fig. S7. **Quantification of endogenous CO in tissues.** The amounts of CO contained in the lung, muscle, cerebrum, and cerebellum without (–) and with flushing (+) the organs with saline (200 mL) to remove residual blood. The amounts of CO were quantified by the hemoCD1 assay. Each bar represents the means ± SE (n = 3–6 rats per group). *p < 0.05 vs –flushing.

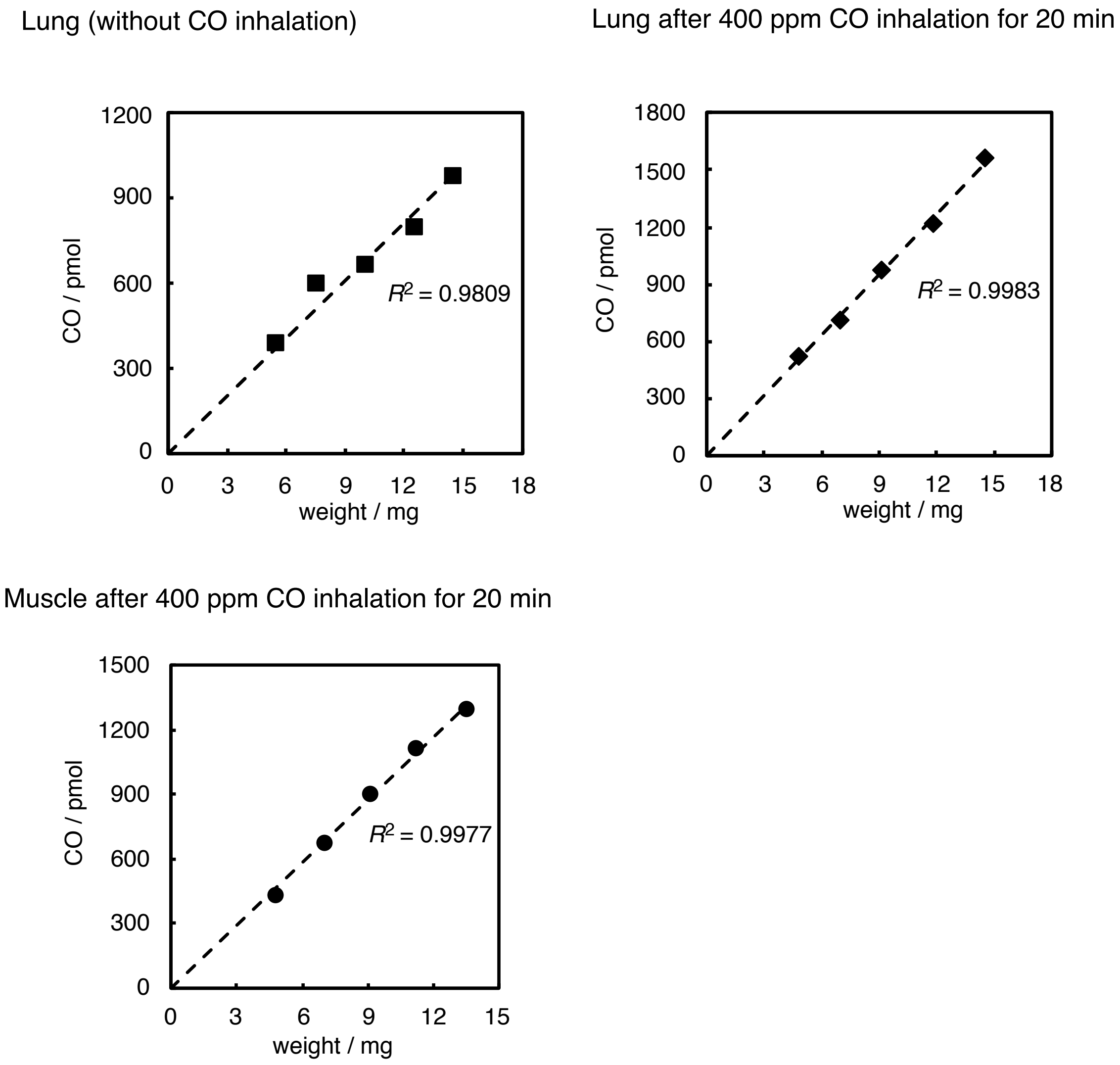

Fig. S8. **Plots of the wet weight of tissues (lung and muscle, with and without CO inhalation) versus the amount of CO detected by the hemoCD1 assay.** The linearity ensures accuracy of the CO quantification assay.

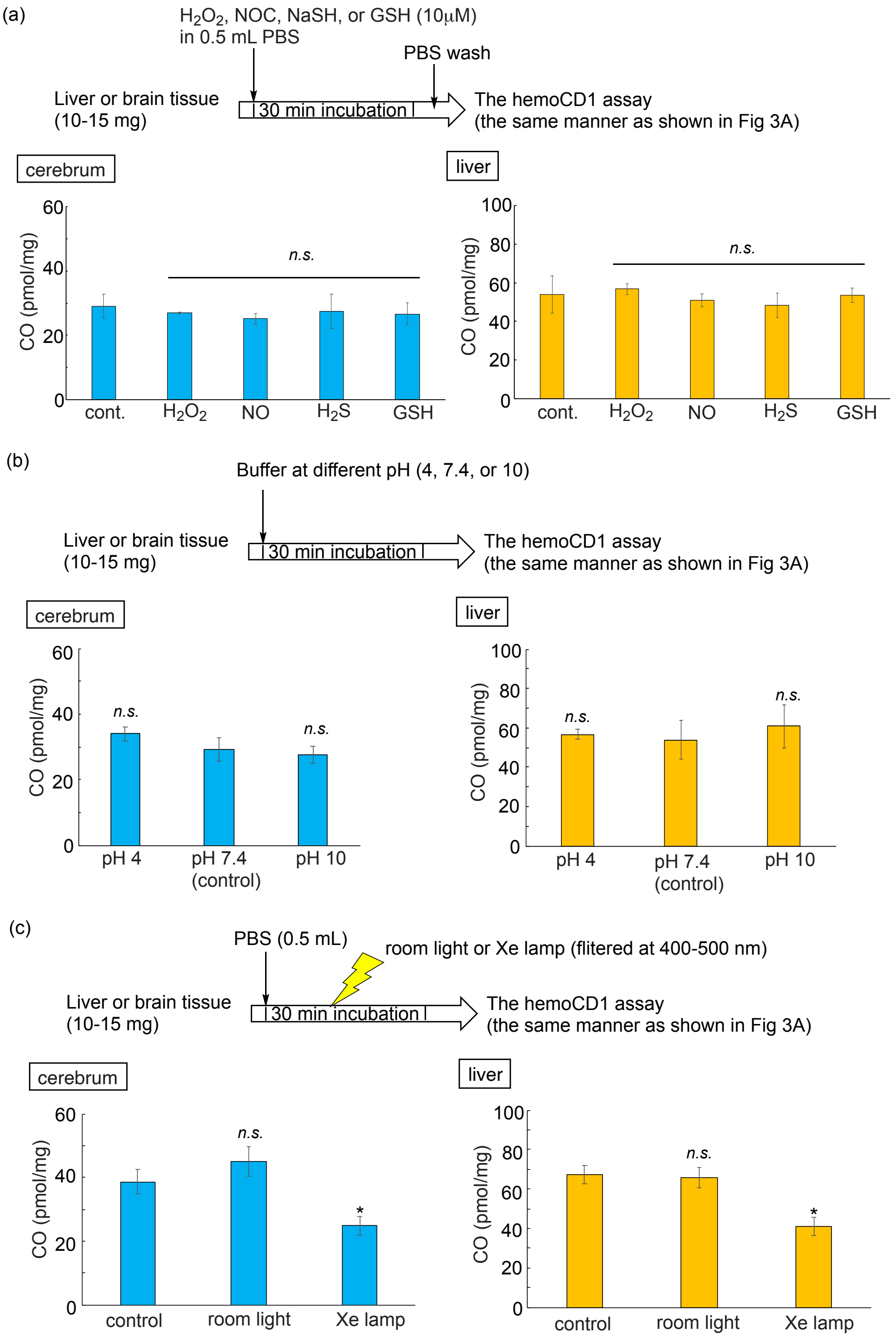

Fig. S9. Effects of external reactive species (a), pH (b), and light (c) on quantification of endogenous CO in the liver and brain tissues by hemoCD1. The data indicate that the assay using hemoCD1 was unaffected by the reactive species, H_2O_2, NO, H_2S and GSH, pH (from 4 to 10), and room light. The data in c indicate that the strong irradiation by Xe lamp reduced CO due to dissociation of the CO-Fe complex. Statistical significance, *$p < 0.05$ vs control; not significant, *n.s.*

a)

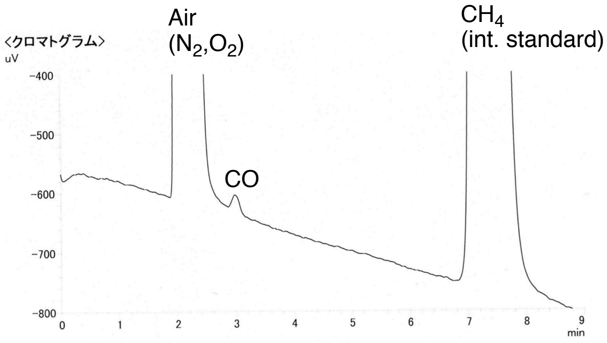

b)

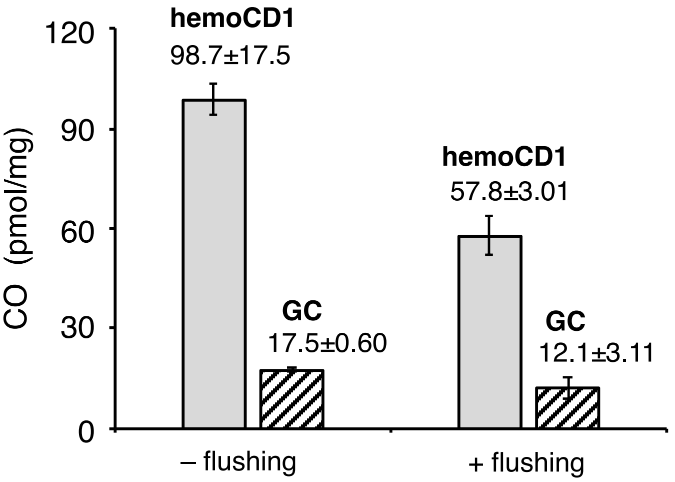

Fig. S10. **Comparison between the hemoCD1 assay and the gas chromatography (GC) method for CO quantification in tissues.** (a) A typical gas chromatogram recording the amount of CO from a liver sample. The headspace gas was analyzed by a TCD detector as reported in the literature, (see Ref 16 in the text). (b) The amounts of CO in the liver tissues (– or + flushing) quantified by the hemoCD1 assay and the GC method. Each bar represents mean ± SE (n = 3–6).

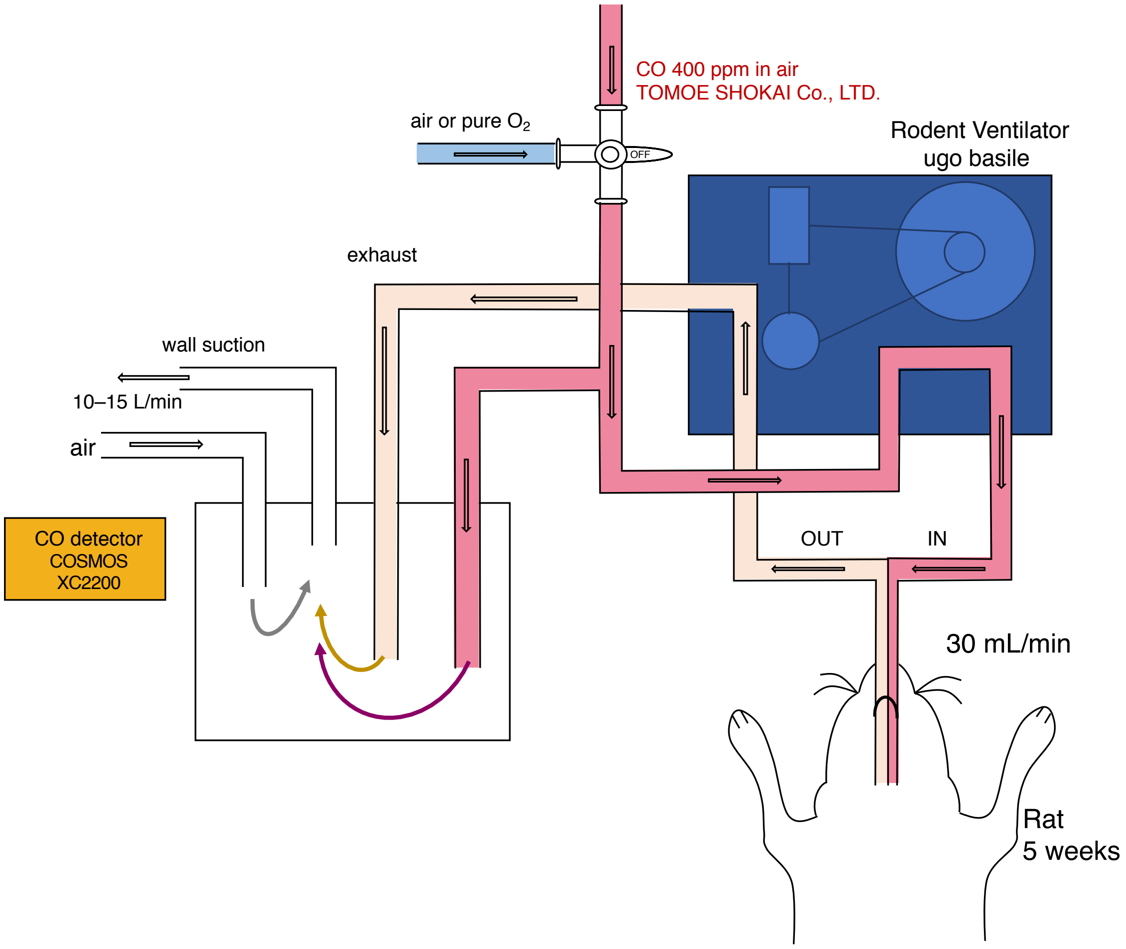

Fig. S11. Schematic representation of the experimental setup for CO gas inhalation in rats. The apparatus allowed a rapid switch from inhaled CO gas (400 ppm) to air or O_2 by handling the three-way stop cock. The rate of inhalation was controlled by the rodent ventilator. The exhaled gas was mixed with air before exiting. For safety reasons, a CO detector was used for monitoring the atmospheric concentrations of CO.

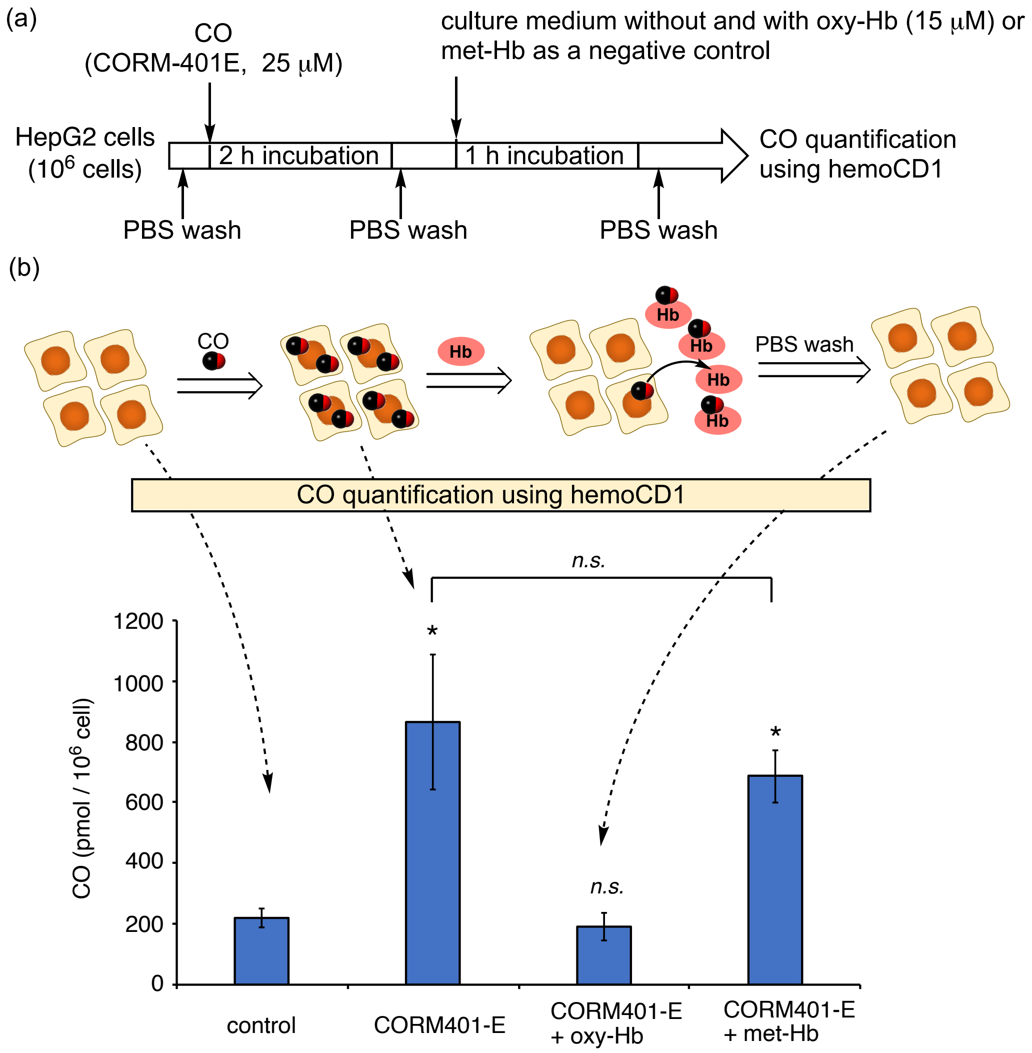

Fig. S12. *In vitro* experiments to demonstrate CO transfer from tissues to Hb. (a) Hepatocytes (1 x 10^6) were treated for 2 h with 25 µM CORM-401E, a CO-releasing molecule recently synthesized in our laboratory for efficient intracellular CO delivery tool (unpublished data, patent pending: PCT/JP2020/006033). Cells were washed out and then treated with medium in the presence of oxy-Hb. After removing the medium containing Hb, the amount of residual CO in cells was quantified by the hemoCD1 assay. Met-Hb, which does not bind CO, was used as a negative control. (b) The amounts of CO in cells determined by the hemoCD1 assay at each step. The results show that cells incubated with CORM401-E accumulated intracellular CO, which was then transferred to oxy-Hb added to the medium. In contrast, CO levels remained high in cells incubated with CORM401-E followed by addition of met-Hb. Each bar represents mean ± SE (n = 3). Statistical significance, *$p < 0.05$; not significant, *n.s.* versus control.

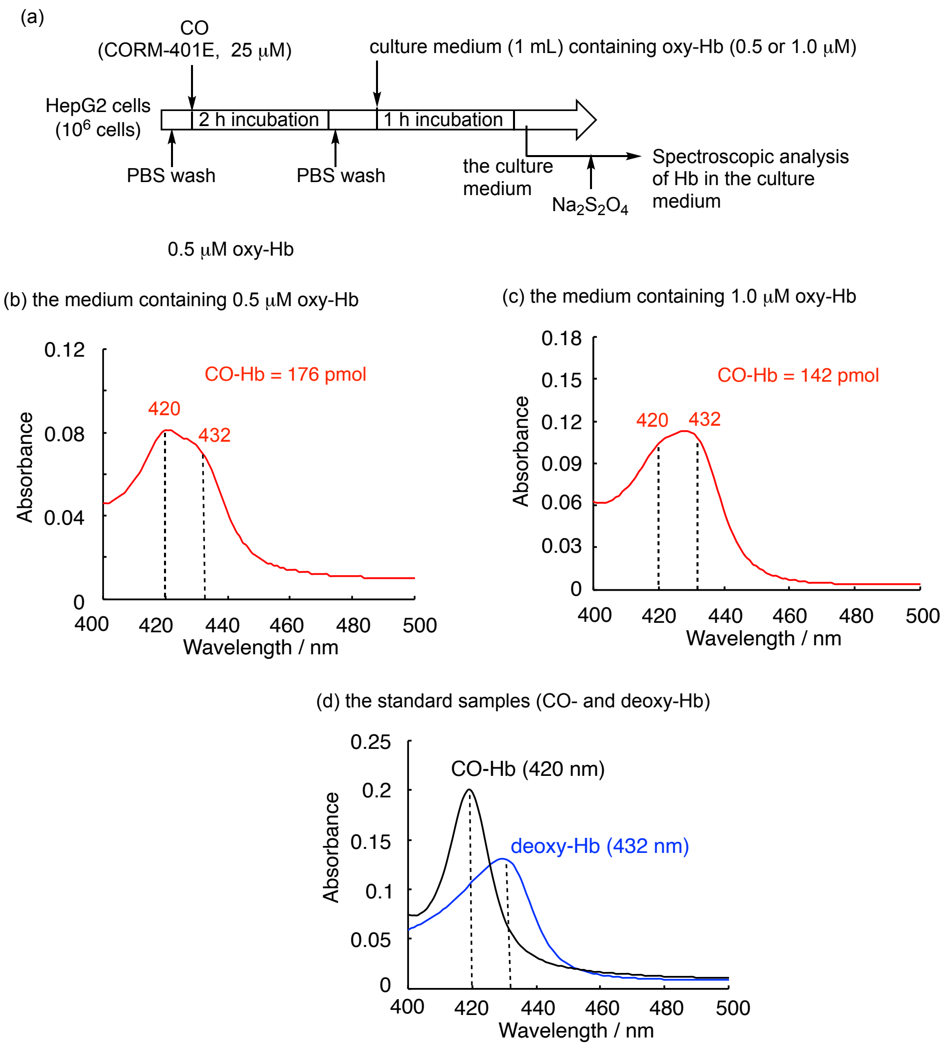

Fig. S13. *In vitro* experiments to monitor CO transfer from cells to Hb in medium. (a) The experimental flowchart is identical to that shown in Figure S12, except for the concentration of oxy-Hb (0.5 and 1.0 μM) added in the medium. The concentrations were adjusted to monitor the content of CO-Hb in the medium. (b,c) The UV-vis spectra of the culture medium containing Hb where $Na_2S_2O_4$ was added before the measurements. (d) The standard spectra of CO- and deoxy-Hb (1 μM each). From the spectra, CO-Hb (420 nm) was found in the medium as a result of CO transfer from the cell to the medium containing oxy-Hb. Based on the absorbances at 420 and 432 nm, and these molar coefficients (F. L. Rodkey et al., *Clin. Chem.* **1979**, *25*, 1388–1393), the amount of CO-Hb in the medium were determined to be 176 and 142 pmol in the presence of 0.5 and 1.0 μM Hb in media, respectively. As the values were somewhat smaller than those measured by the hemoCD1 assay (see Figure S12), we presume that some CO is lost from CO-Hb during the experimental handling.

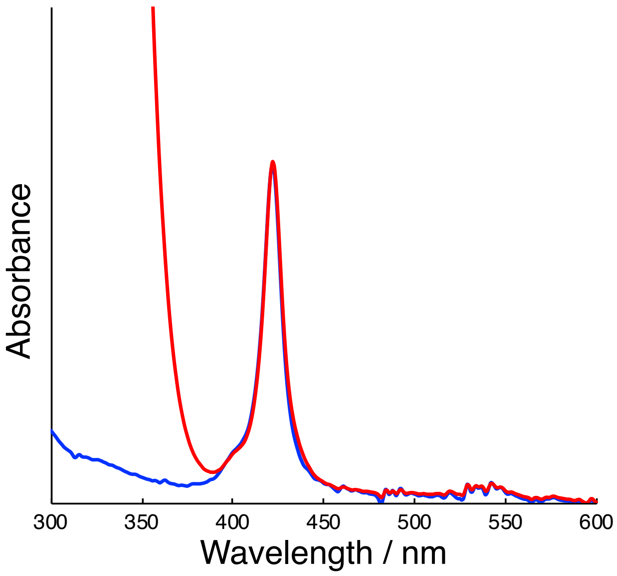

Fig. S14. **UV-vis spectra of rat urine collected after i.v. infusion of oxy-hemoCD1 to CO-treated rats before (blue) and after the addition of $Na_2S_2O_4$ (red).** The completely overlapped spectral shapes indicates that hemoCD1 in the urine was almost 100% in the CO-bound form. A similar spectral profile has been previously reported by us (Refs 35,36).

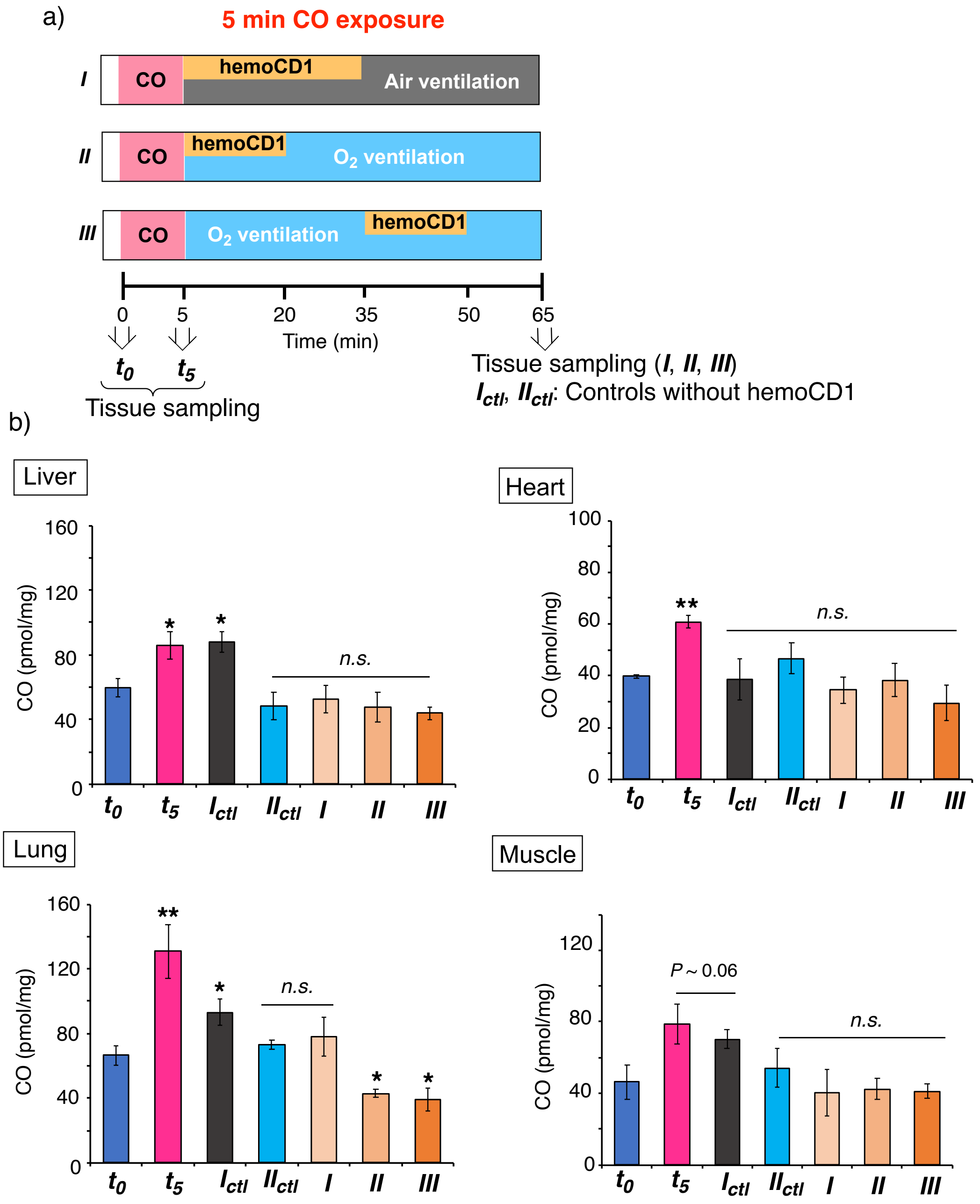

Fig. S15. Effect of air/O_2 ventilation in combination with hemoCD1 on CO distribution in tissues after CO inhalation *in vivo*. (a) Experimental protocols used are the same as those in Fig. 7A. (b) Amounts of CO detected in liver, heart, lung and muscle tissues collected as indicated in (a). Each bar represents the mean ± SE (n = 3–6). Statistical significance, *$p < 0.05$, **$p < 0.01$; not significant, *n.s.* versus $\boldsymbol{t_0}$.

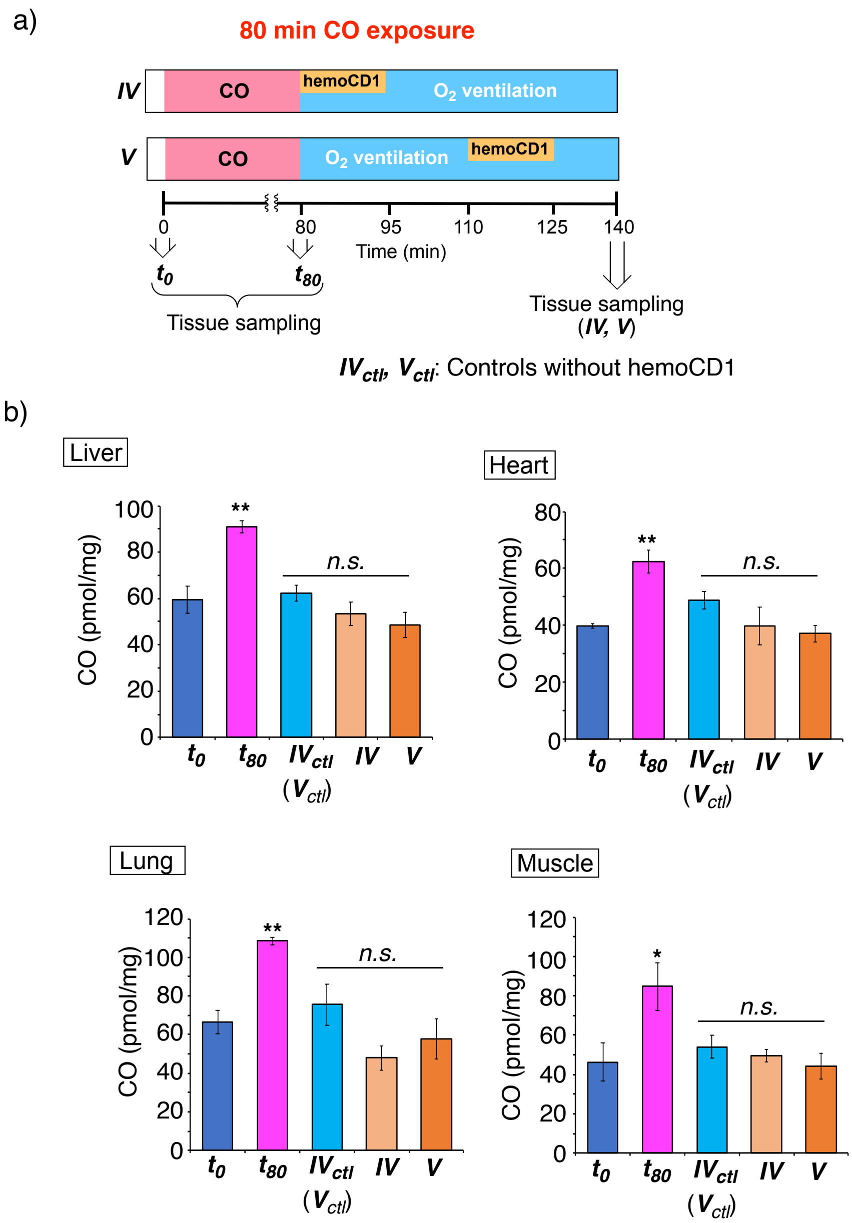

Fig. S16. **Effect of O_2 ventilation in combination with hemoCD1 on CO distribution in tissues after CO inhalation *in vivo*.** Experimental protocols used are the same as those in Fig. 7B. (b) Amounts of CO detected in liver, heart, lung and muscle tissues collected as indicated in (a). Each bar represents the mean ± SE (n = 4–6). Statistical significance, *$p < 0.05$, **$p < 0.01$; not significant, *n.s.* versus $\boldsymbol{t_0}$.
