# Supplementary material for: Sensitive quantification of carbon monoxide in vivo reveals a protective role of circulating hemoglobin in CO intoxication
Source: Commun Biol. 2021 Mar 29;4:425. doi: 10.1038/s42003-021-01880-1 (PMC8007703; doi:10.1038/s42003-021-01880-1)
Supplement: Supplementary file 3 — Descriptions of Additional Supplementary Files [file 42003_2021_1880_MOESM3_ESM.pdf]

Descriptions of Additional Supplementary Files

File name: Supplementary Data 1
Description: Source data for Figures 3–7.

File name: Supplementary Data 2
Description: Source data for Figures S6, 7, 8, 9, 10, 12, 15, and 16.
