# Supplementary material for: Sensitive quantification of carbon monoxide in vivo reveals a protective role of circulating hemoglobin in CO intoxication
Source: Commun Biol. 2021 Mar 29;4:425. doi: 10.1038/s42003-021-01880-1 (PMC8007703; doi:10.1038/s42003-021-01880-1)
Supplement: Supplementary file 6 — Reporting Summary [file 42003_2021_1880_MOESM6_ESM.pdf]

nature research

Corresponding author(s): Hiroaki Kitagishi

Last updated by author(s): Jan 19, 2021

Reporting Summary

Nature Research wishes to improve the reproducibility of the work that we publish. This form provides structure for consistency and transparency in reporting. For further information on Nature Research policies, see our Editorial Policies and the Editorial Policy Checklist.

Statistics

For all statistical analyses, confirm that the following items are present in the figure legend, table legend, main text, or Methods section.

n/a	Confirmed	
☐	☒	The exact sample size (*n*) for each experimental group/condition, given as a discrete number and unit of measurement
☐	☒	A statement on whether measurements were taken from distinct samples or whether the same sample was measured repeatedly
☐	☒	The statistical test(s) used AND whether they are one- or two-sided<br>*Only common tests should be described solely by name; describe more complex techniques in the Methods section.*
☐	☒	A description of all covariates tested
☐	☒	A description of any assumptions or corrections, such as tests of normality and adjustment for multiple comparisons
☐	☒	A full description of the statistical parameters including central tendency (e.g. means) or other basic estimates (e.g. regression coefficient) AND variation (e.g. standard deviation) or associated estimates of uncertainty (e.g. confidence intervals)
☐	☒	For null hypothesis testing, the test statistic (e.g. *F*, *t*, *r*) with confidence intervals, effect sizes, degrees of freedom and *P* value noted<br>*Give P values as exact values whenever suitable.*
☒	☐	For Bayesian analysis, information on the choice of priors and Markov chain Monte Carlo settings
☒	☐	For hierarchical and complex designs, identification of the appropriate level for tests and full reporting of outcomes
☒	☐	Estimates of effect sizes (e.g. Cohen's *d*, Pearson's *r*), indicating how they were calculated

Our web collection on statistics for biologists contains articles on many of the points above.

Software and code

Policy information about availability of computer code

Data collection	Data were collected by NanoPhotometer C40, Shimadzu UV-2450 spectrometer, Shimadzu GC-2014, and blood gas analyzer ABL825.
Data analysis	Data were organized and analyzed using a Microsoft Excel software.

For manuscripts utilizing custom algorithms or software that are central to the research but not yet described in published literature, software must be made available to editors and reviewers. We strongly encourage code deposition in a community repository (e.g. GitHub). See the Nature Research guidelines for submitting code & software for further information.

Data

Policy information about availability of data

All manuscripts must include a data availability statement. This statement should provide the following information, where applicable:

- Accession codes, unique identifiers, or web links for publicly available datasets
- A list of figures that have associated raw data
- A description of any restrictions on data availability

All data are present in the paper and the Supplementary Materials. All raw and processed data will be made available upon request

Field-specific reporting

Life sciences study design

All studies must disclose on these points even when the disclosure is negative.

Sample size	For experiments involving quantification of CO, n=3 was chosen as the minimal replicate number.
Data exclusions	No data excluded.
Replication	All replication experiments were confirmed successful.
Randomization	The tissue samples from rats were taken and selected randomly in the measurement.
Blinding	The collection and analysis of the tissue samples were independently performed, and the analysis was partly conducted with blinding.

Reporting for specific materials, systems and methods

We require information from authors about some types of materials, experimental systems and methods used in many studies. Here, indicate whether each material, system or method listed is relevant to your study. If you are not sure if a list item applies to your research, read the appropriate section before selecting a response.

Materials & experimental systems

n/a	Involved in the study
☒	☐ Antibodies
☒	☐ Eukaryotic cell lines
☒	☐ Palaeontology and archaeology
☐	☒ Animals and other organisms
☒	☐ Human research participants
☒	☐ Clinical data
☒	☐ Dual use research of concern

Methods

n/a	Involved in the study
☒	☐ ChIP-seq
☒	☐ Flow cytometry
☒	☐ MRI-based neuroimaging

Animals and other organisms

Policy information about studies involving animals; ARRIVE guidelines recommended for reporting animal research

Laboratory animals	Five weeks old Lewis and Sprague-Dawley rats were purchased from Charles River Co. Ltd., (Yokohama, Japan). Before undergoing any experiment, animals were acclimatized for a week under air conditioning at 26 ± 0.5°C with access to water and food ad libitum.
Wild animals	No wild animals were used.
Field-collected samples	No field collected samples were used.
Ethics oversight	The animal experiments approved by the Institutional Review Board of Tokai University and the Guidelines for Animal Experiments of Doshisha University. Animals received humane care as required by the institutional guidelines for animal care and treatment in experimental investigations according to the Guide for the Care and Use of Laboratory Animals (Institute of Laboratory Animal Resources, 1996).

Note that full information on the approval of the study protocol must also be provided in the manuscript.
